# IP-10 Can Be Measured in Dried Plasma Spots in Patients with Chronic Hepatitis C Infection

**DOI:** 10.1371/journal.pone.0045181

**Published:** 2012-09-14

**Authors:** Morten Ruhwald, Ellen Sloth Andersen, Peer Brehm Christensen, Belinda Klemmensen Moessner, Nina Weis

**Affiliations:** 1 Clinical Research Centre, Copenhagen University Hospital, Hvidovre, Copenhagen, Denmark; 2 Department of Infectious Diseases, Copenhagen University Hospital, Hvidovre, Copenhagen, Denmark; 3 Department of Infectious Diseases, Copenhagen University Hospital, Rigshospitalet, Copenhagen, Denmark; 4 Department of Infectious Diseases, Odense University Hospital, Odense, Denmark; 5 Faculty of Health Sciences, Copenhagen University, Copenhagen, Denmark; Institut Pasteur, France

## Abstract

The chemokine IP-10 (CXCL10) is a candidate marker for hepatitis C virus (HCV) fibrosis monitoring. The aim of this proof-of-concept study is to assess if IP-10 measurements from dried plasma spots (DPS) are accurate in HCV-infected patients with either minimal or significant fibrosis. We measured IP-10 levels in plasma and DPS of 21 HCV-infected patients with cirrhosis and 19 patients with no/little fibrosis (determined with FibroScan). Cirrhotic patients had significantly higher levels of IP-10 compared to patients with minimal fibrosis. DPS and plasma measurements of IP-10 are comparable and the correlation was excellent (r^2^ = 0.97, p<0.0001). The DPS based method for IP-10 detection performs well in HCV-infected patients with either minimal or significant fibrosis.

## Introduction

Globally, 170 million individuals are chronically infected with Hepatitis C virus (HCV). The hallmark of HCV infection is the progressive development of liver fibrosis, leading to liver cirrhosis and potentially hepatocellular carcinoma. The level of liver fibrosis predicts liver related complications and therefore the assessment of liver fibrosis is a cornerstone in the management of patients chronically infected with HCV. Each year 0.5 million people die of HCV-related diseases.

For the last fifty years, liver biopsy has been considered the gold standard for fibrosis and cirrhosis assessment, but recent reports indicate that biopsy does not fulfill the requirements of a surrogate marker; mainly because of its high complication and sampling error rate, high inter- and intra observer variability, cost and patient reluctance to undergo serial monitoring [1], [2]. In the last decade, several promising non-invasive alternatives have emerged. Liver stiffness measurement using FibroScan (Echosens, Paris, France) is a rapid method with high accuracy for the monitoring of HCV induced fibrosis and cirrhosis [1]. Also blood sample tests, such as the FibroTest (Biopredictive, Paris, France), have been shown to have a good correlation with advanced liver fibrosis.

There are limitations to the novel non-invasive tools. The FibroScan is expensive to acquire and the blood tests rely on accurate measurement using multiple assays. These tests are therefore only offered in a few, validated laboratories. As these promising novel modalities will most likely not reach the large majority of HCV infected patients in the developing world, simpler and cheaper alternatives are needed.

A series of reports have demonstrated that chemokine Interferon-γ Inducible protein 10 (IP-10, CXCL10) is a promising single marker correlate for liver fibrosis, and an IL-28b independent negative predictor of treatment outcome in HCV infected patients[3]–[7]. IP-10 is a key driver in both innate and antigen specific immune responses by directing Th1 cells to the site of inflammation [8], [9]. IP-10 is secreted by HCV infected hepatocytes into the blood and can therefore be seen as a direct proxy of ongoing inflammation in the liver [10], [11]. Recently, it was shown that in patients with chronic HCV infection the majority of plasma IP-10 exists in a 2 amino-acid truncated antagonist form, which inhibits the desired antiviral effects of IP-10 and could play an important role in pathology [12]. Compared to most of the key pro-inflammatory T cell cytokines (e.g. IFN-γ), IP-10 is expressed in 100 fold higher levels making it easy to measure also with simple technology [13].

Drying of plasma and blood on filter paper is a reliable method for conserving proteins. The method is state-of-art in national screening programs of neonates and it enables very simple sample acquisition (e.g. a finger or heel prick), and safe and cheap long distance transport using normal mail service [14]. Recent publications have demonstrated that dried blood spots (DBS) are a reliable alternative to serum specimens for detecting anti-HCV, quantifying HCV RNA and genotyping HCV [15]. We have recently developed an ELISA based assay for IP-10 detection in DBS and dried plasma spots (DPS) [16]. Using this assay we have demonstrated a very high correlation between DPS, DBS and plasma IP-10 levels in sample from healthy donors and patients with *M.tuberculosis* infection, and that this method renders comparable diagnostic accuracy as the current state-of-art diagnostic assay for infection with *M.tuberculosis*, the Quantiferon test (Aabye et al unpublished). It is unknown how the filter paper based method for IP-10 detection performs in samples from patients with chronic HCV infection. The aim of this study was to assess if the filter paper based method for IP-10 detection compares to IP-10 detected in plasma from HCV infected patients with either minimal or significant liver fibrosis.

## Materials and Methods

### Ethics Statement

The study has been approved by the Danish National Committee on Biomedical Research Ethics (H-D-2007-0087) in accordance with the Helsinki Declaration. All patients had given written and oral consent to participate in the study.

### Patient Material

A detailed description of the patients included has previously been published [17]. In brief, we included 40 patients with HCV genotype 1 infection. Twenty-one had cirrhosis and 19 had no/mild fibrosis. Transient elastography (FibroScan®, medium probe, software version 1.30) was used for diagnosing the stage of fibrosis. Liver stiffness measurements were considered to be successful if ten valid measurements were obtained (valid measurements >60% and interquartile range <25%). Patients were diagnosed as having no/mild fibrosis when the liver stiffness was below 7.7 kPa, and as having cirrhosis when the values were equal to or above 13.0 kPa. Patients with hepatocellular carcinoma, previous liver transplantation or co-infection with other HCV genotypes than 1, hepatitis B virus or HIV were excluded. Compared to patients with no/mild fibrosis, the patients in the cirrhotic group were older (median age was 57 vs. 46 years, p<0.0001), predominately male (15/21 (71%) vs. 8/19 (42%), p = 0.117) and had a higher body mass index (median 26.5 kg/m^2^ (inter quartile range (IQR) 23.9–29.9 kg/m^2^) vs. 22.6 kg/m^2^ (IQR 20.4–26.1 kg/m^2^), p = 0.019).

### Sample Preparation

Blood was drawn on the day of FibroScan and EDTA plasma was stored at -80°C until analysis. Plasma samples were thawed and IP-10 concentration was determined with ELISA. DPS samples were prepared as described in detail elsewhere [16], in brief 25 µl plasma was added to filter paper (903 Protein Saver™ cards,Whatman) in duplicates. After 4 hours drying on the lab bench, DPS samples were stored in gas-impermeable plastic bags with a desiccator at room temperature for 7 days before analysis.

### IP-10 Measurement in Plasma and DPS with ELISA

IP-10 levels in plasma and DPS samples were determined with an ELISA based assay, developed and optimized for the monitoring of IP-10 in plasma and filter paper samples [16]. In brief, we made rat and murine hybridoma cell lines producing monoclonal antibodies (mAbs) specific for IP-10. Maxisorb plates (Nunc, Denmark) were coated with murine mAb in carbonate buffer, washed, blocked and dried for later use. On day of assay 20 µl of each plasma sample was diluted 5 times in assay buffer with HRP-conjugated detection mAb. When used for DPSs, 2 discs of 5.5 mm were punched from the centre of the DPS using a standard office paper puncher (Impega, UK) and incubated with 100 µL assay buffer. Plasma samples and DPS discs were incubated for 2 hours at room temperature (23°C) and washed x 3. HRP-substrate (TMB One, Trichem) was added, plates were revealed for 30 minutes before colour reaction was stopped with 100 µL H_2_SO_4_ and absorbance was read. Concentrations were calculated using a standard curve with a linear range from 2.5–600 pg/ml (Peprotec, USA). Plasma concentrations were corrected for the dilution factor (multiplied x5), DPS samples are presented as pg/2 discs.

### Statistics

IP-10 concentrations were compared with non-parametric methods (Mann Whitney U test and Spearman correlation). Data were plotted and analysed using GraphPad Prizm 5.0 for Mac OS X (GraphPad, USA).

### Ethical

The study has been approved by the Danish National Committee on Biomedical Research Ethics (H-D-2007-0087) in accordance to the Helsinki Declaration. All patients gave written and oral consent to participate in the study.

## Results

**Figure 1:**
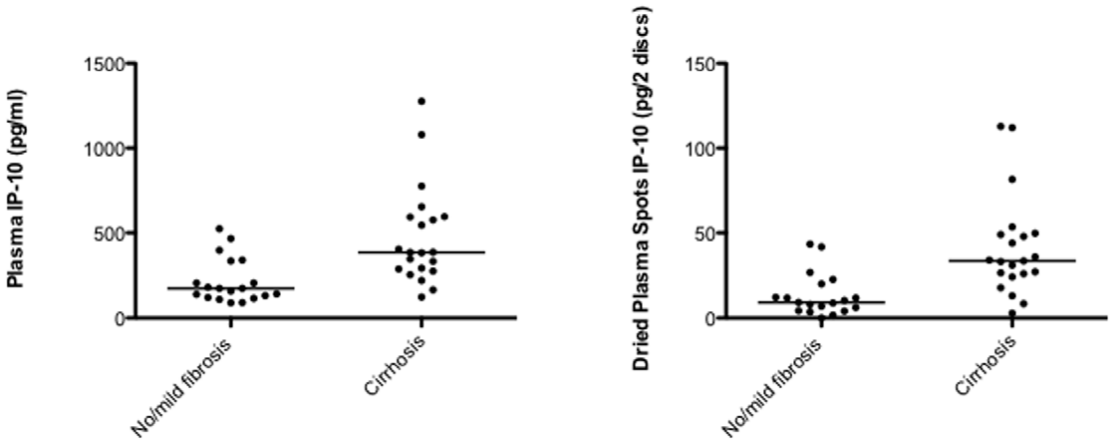
IP-10 measurements. IP-10 measured in plasma (left) and dried plasma spots (right) from 19 patients with chronic hepatitis C infection and no/mild liver fibrosis and 21 patients with liver cirrhosis. Line denote median. There was a significant difference between the groups in both plasma and dried plasma spots, p<0.0004.

**Figure 2:**
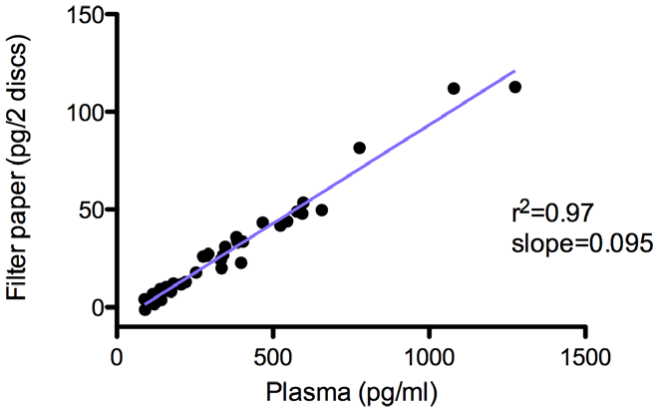
Correlation between IP-10 detected in plasma and dried plasma spots. Samples from 19 patients with chronic hepatitis C infection and no/mild liver fibrosis and 21 patients with liver cirrhosis were compared. There was a highly significant correlation between IP-10 detected with the two methods (r^2^ = 0.97, p<0.0001).

**Figure 3:**
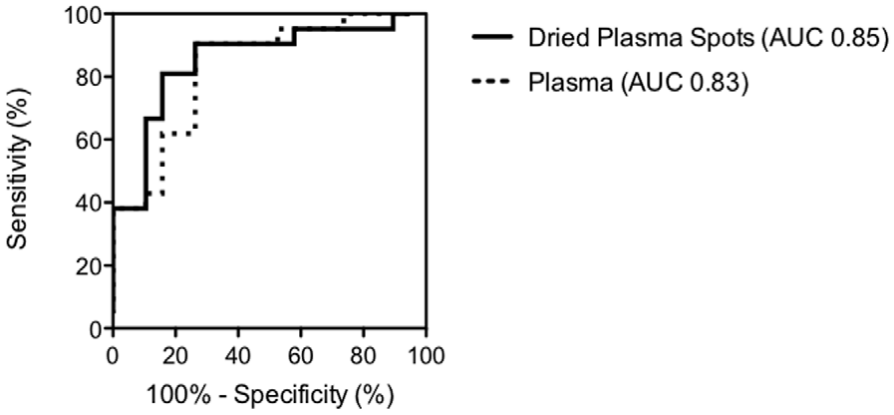
A comparison of Receiver Operation Characteristic Curves of IP-10 determined in dried plasma spots and plasma. Analysis included samples from 21 patients with cirrhosis (regarded as cases) and 19 patients with no/mild fibrosis (regarded controls in the analysis). The Area Under the Curve reflects the markers’ ability to differentiate between the two groups of patients. There was no significant difference between the two methods.

Plasma samples from patients with cirrhosis had significantly higher levels of IP-10 median 385 pg/ml (IQR 282–595 pg/ml)) compared to patients with no/mild fibrosis 174 pg/ml (IQR 120–335 pg/ml, p<0.0001, Figure 1). Similar differences were found in the DPS samples median 34 pg/2 DPS discs (IQR 25–49 pg/2 DPS discs)) compared to 9pg/2 DPS discs (IQR 4–20 pg/2 DPS discs, p<0.0001). There was an excellent correlation between plasma and DPS samples (r^2^ = 0.97, p<0.0001, Figure 2). ROC curve analysis revealed a comparable discriminatory capability between the two methods AUC 0.82 and 0.85 for IP-10 determined in plasma and DPS samples, respectively (Figure 3).

## Discussion

In this proof-of-concept study we assess whether IP-10 can be measured in HCV-infected patients by the DPS method. We show that IP-10 determined in plasma and DPS samples have excellent correlation and comparable discriminatory capability between patients with no/mild fibrosis and patients with cirrhosis.

Plasma levels of IP-10 have proven to be a valid correlate for HCV fibrosis [18], a measure of chronic HCV disease activity [19], and an IL-28b independent negative predictor of treatment effect[4]–[7], [11], [19]. We have previously developed and validated the filter paper method for IP-10 detection in DBS and DPS samples, and we have shown that the recovery and stability of IP-10 in filter paper samples is very high and comparable to IP-10 detected in plasma (r^2^>0.97). The range of the assay allows for accurate detection of both the levels of IP-10 found in the blood, and the high levels seen after in-vitro stimulation of whole blood with disease specific antigens and mitogens ([16] and Aabye et al. unpublished). This report supports our previous work in terms of technical performance of the filter paper IP-10 method and extends the usefulness IP-10 detection in un-stimulated patient samples.

Liver biopsy, and to some extend FibroScan and FibroTest, are surrogate markers for fibrosis in liver disease. In contrast, the plasma level of IP-10 appears a more dynamic and functional marker, directly linked to interplay of virus and immune response [10], [11]. IP-10 appears to reflect both the extent of liver fibrosis as well as the immune activity (e.g. IP-10 increase during a flare-up and after interferon treatment in responsive patients [11], [12], [18]). Further prospective studies, with sequential measurements of IP-10, are needed to assess the contribution of these two potential components in the total plasma IP-10 level, and to explore if this can be used to guide patient management.

Limitations: In this study we use plasma and not whole blood. Whole blood from a finger prick would be the optimal and simplest source of material for the filter paper application if this method should prove valid for clinical use. We have previously demonstrated that IP-10 levels detected in DPS discs are comparable to DBS discs in terms of signal intensity (r^2^ = 0.99; regression slope = 1.01, p<0.0001) and IP-10 stability [16], wherefore we think that our DPS results can be transferred to DBS analysis. We use FibroScan as a proxy for biopsy and histological classification of fibrosis, FibroScan readings can differ from biopsy results and the data should be interpreted in this light [20]. However, of the 40 patients included, ten had within 5 years of inclusion undergone a percutaneous liver biopsy of which, 9 (90%) had a fibrosis stage determined by liver biopsy in agreement with the fibrosis stage determined by FibroScan [17]. Another limitation is the comparison of the two highly selected groups of HCV genotype 1 infected patients and that the two groups were not matched with respect to age, gender or body mass index. This comparison of the least and the most affected within a strictly defined disease entity is artificial, and can lead to over interpretation of the associations observed. Nevertheless, a clear case-control design is essential, in the early stages of validating a new biomarker or a new method such as the DPS/DBS method; if no association is found, then the biomarker or method should probably not be explored further [21]. Future studies including patients representing intermediate degrees of fibrosis are needed to define cut offs and establish correlates between plasma levels of IP-10 and other surrogate markers of fibrosis. In addition the investigations of IP-10 as marker of fibrosis must be extended to patients infected with other genotypes than 1. Finally the median levels in the no/mild fibrosis detected with the DPS method were only 4 fold higher than the lower limit of detection for the assay [16]. This could compromise the ability to accurately compare uninfected controls to patients with no/low fibrosis, but not when comparing patients infected with HCV with varying degree of fibrosis.

Regardless of the limitations in this study, it appears from this and other recent studies that the filter paper method is as reliable as a plasma sample for detection of chemokines such as IP-10 [16], [22]. Given that IP-10 can be demonstrated to be a valid fibrosis monitoring- and clinical decision tool in well-powered prospective studies, the filter paper based method opens new possibilities for HCV screening and monitoring, especially in combination with filter paper based HCV RNA quantification and genotyping [15]. IP-10 is readily detectable in DBS as in DPS, which makes sampling as simple as a home blood glucose monitoring [14], and could enable a new approach to HCV disease monitoring, based on frequent at-home testing. Automated high-throughput filter paper punching and analysis equipment is already standard for neonatal screening and can be adapted for IP-10 measurement to centralize analysis and reduce analytical imprecision.

In conclusion, this study we establish that IP-10 levels in plasma can be detected after the plasma has been dried on filter paper, but more studies are needed to substantiate IP-10 as a valid biomarker for liver fibrosis.
